# Effects of Mobilization with Movement Versus Sham Mobilization on Inflammatory Biomarkers and Clinical Outcomes in Patients with Knee Osteoarthritis: A Randomized Double-Blind Clinical Trial

**DOI:** 10.3390/medicina62102016

**Published:** 2026-10-18

**Authors:** Abdulraouf H. Ayoub, Mohammed Z. Muslem, Mohamed M. Ahmed, Amani A. Al Aqeel, Zenat A. Khired, Mahdi M. Alnajai, Ali M. Alshami

**Affiliations:** 1 Department of Physical Therapy, College of Applied Medical Sciences, Imam Abdulrahman Bin Faisal University, Dammam 31441, Saudi Arabia; 2 Physical Therapy Department, Jazan University Medical City Hospital, Jazan University, Jazan 82817, Saudi Arabia; 3 Department of Physical Therapy, College of Nursing and Health Sciences, Jazan University, Jazan 82817, Saudi Arabia; 4 Department of Surgery, College of Medicine, Jazan University, Jazan 82817, Saudi Arabia; 5 Laboratory and Blood Bank Department, Jazan University Medical City Hospital, Jazan University, Jazan 82817, Saudi Arabia

**Keywords:** knee osteoarthritis, mobilization with movement, sham mobilization, inflammatory biomarkers, C-reactive protein, erythrocyte sedimentation rate

## Abstract

*Background and Objectives:* Low-grade systemic inflammation plays an important role in the pathophysiology of knee osteoarthritis (OA), yet the effects of joint mobilization on inflammatory biomarkers remain unclear. This randomized clinical trial investigated the effects of mobilization with movement (MWM) compared with sham MWM on inflammatory biomarkers and clinical outcomes in individuals with knee OA. *Materials and Methods:* Forty participants with mild-to-moderate knee OA were randomly assigned to either a mobilization group receiving standardized care plus MWM (*n* = 20) or a sham mobilization group receiving standardized care plus sham MWM (*n* = 20). Interventions were delivered twice weekly for four weeks. Primary outcomes were inflammatory biomarkers, including C-reactive protein (CRP) and erythrocyte sedimentation rate (ESR). Secondary outcomes included pain intensity (Visual Analog Scale [VAS]), physical function (Western Ontario and McMaster Universities Osteoarthritis Index [WOMAC]), knee range of motion (ROM), and muscle strength. Outcomes were assessed at baseline and after the 4-week intervention. This study was prospectively registered at ClinicalTrials.gov (NCT07166068) on 28 November 2024. *Results:* Both groups demonstrated significant improvements in one inflammatory biomarker, namely CRP, and all secondary outcomes following treatment (all *p* < 0.05). However, no significant between-group differences were observed for CRP, ESR, pain, function, or muscle strength (all *p* ≥ 0.256). Participants in the MWM group showed greater improvement in knee flexion ROM compared with the sham MWM group (adjusted mean difference = 6.56°, 95% CI: 0.14–13.00, *p* = 0.046), with a moderate effect size. *Conclusions:* MWM combined with standardized care did not produce superior improvements in inflammatory biomarkers or most clinical outcomes compared with sham MWM over four weeks.

## 1. Introduction

Knee osteoarthritis (OA) is one of the most common degenerative joint disorders affecting middle-aged and older adults worldwide [1], with an estimated 364.58 million prevalent cases globally in 2019 [2]. In Saudi Arabia, the overall prevalence of symptomatic knee OA was reported to be 18.9% among adults [3]. Individuals with knee OA commonly experience pain, stiffness, and reduced mobility [4], which contribute to decreased quality of life and increased healthcare burden [5].

Osteoarthritis has been traditionally understood as a mechanical wear-and-tear condition [6]. However, current evidence identifies it as a multifactorial disease involving biomechanical, metabolic, and low-grade inflammatory processes [7]. Low-grade systemic inflammation plays a central role in cartilage degeneration through cytokines such as interleukin-1β (IL-1β), interleukin-6 (IL-6), and tumor necrosis factor-α (TNF-α), which stimulate degradative enzymes including matrix metalloproteinases (MMPs) and aggrecanases (ADAMTS), leading to extracellular matrix breakdown [8]. Systemic biomarkers such as C-reactive protein (CRP) and erythrocyte sedimentation rate (ESR) are commonly used to reflect inflammatory activity in knee OA, with higher levels associated with increased pain and radiographic severity [9,10].

Physical therapy is strongly endorsed across major international guidelines as a first-line, non-pharmacological intervention for knee OA, with exercise-based programs identified as the core component of management. These guidelines also support the use of manual therapy to address stiffness, restore joint mobility, and enhance movement efficiency [11,12]. Beyond clinical symptom improvement, emerging evidence indicates that physical therapy interventions may influence systemic inflammatory activity in knee OA, with exercise therapy shown to decrease CRP [13].

Beyond exercise-based interventions, several studies have examined the effects of different physical therapy modalities on inflammatory biomarkers in knee OA. For example, one study reported that electrotherapy significantly reduced ESR levels [14], while another demonstrated that acupuncture produced statistically significant reductions in both CRP and ESR [15]. In addition, Maitland’s joint mobilization demonstrated reductions in CRP [16]. These findings indicate that certain physiotherapy modalities can exert measurable biological effects. However, the influence of manual therapy on systemic inflammation remains insufficiently explored. To the best of our knowledge, only one randomized controlled trial has evaluated a manual mobilization technique in this context, showing that Maitland mobilization reduced CRP levels but did not assess ESR [16]. Notably, this study did not investigate mobilization with movement (MWM), despite evidence suggesting that MWM may yield greater improvements in pain and functional outcomes compared with Maitland techniques [17,18].

Although OA inflammation is mainly intra-articular, it has a systemic component: cytokines released by activated chondrocytes and synoviocytes, particularly IL-6, stimulate hepatic production of CRP [8,19]. MWM may influence this pathway through several mechanisms. By restoring pain-free joint mechanics, it may reduce abnormal stress on articular tissues, a recognized trigger of chondrocyte activation and cytokine release, and thereby relieve local inflammation [20,21]. In addition, knee joint mobilization has been shown to enhance descending pain modulation in individuals with knee OA [22], and manual therapy has been shown to modulate inflammatory cytokines [21]. However, direct evidence for these effects in knee OA is limited, and whether MWM produces measurable changes in systemic inflammatory biomarkers remains an open, exploratory question.

Thus, the current study aimed to evaluate the effects of MWM applied to the knee on CRP and ESR levels in individuals with knee OA. It was hypothesized that MWM would produce greater reductions in these biomarkers compared with sham mobilization. Addressing this gap may enhance understanding of the biological effects of MWM and support its clinical application in managing knee OA.

## 2. Materials and Methods

### 2.1. Study Design, Setting, and Ethical Considerations

This study was a randomized, double-blind, parallel-group clinical trial conducted at the Physiotherapy Department of Jazan University Hospital between May 2025 and November 2025. Participants and outcome assessors were blinded to group allocation; the treating therapists could not be blinded because of the nature of the intervention. The trial was prospectively registered at ClinicalTrials.gov (NCT07166068). Ethical approvals were obtained from the Standing Committee for Sabbatical Leaves, Publication, and Research Ethics at Jazan University (REC-46/03/1199; Protocol ID: HAPO-10-Z-001) and the Institutional Review Board of Imam Abdulrahman bin Faisal University (IRB-PGS-2024-03-774). All participants provided written informed consent prior to enrollment. Patients and the public were not involved in the design, conduct, reporting, or dissemination plans of this trial. No important changes were made to the trial methods, outcomes, or analyses after trial commencement. The study was conducted in accordance with the Declaration of Helsinki and adhered to the Consolidated Standards of Reporting Trials (CONSORT) guidelines.

### 2.2. Sample Size Calculation

The sample size was calculated using G*Power software (version 3.1.9.6; University of Düsseldorf, Düsseldorf, Germany). An analysis of covariance (ANCOVA) with two groups and one covariate was selected, with the significance level set at 0.05 and statistical power at 80%. The effect size (Cohen’s f = 0.50) was derived from a previously published study evaluating ESR [23]. Based on these parameters, the required sample size was 34 participants. To account for an anticipated attrition rate of approximately 20%, the final target sample size was increased to 40 participants (20 per group).

### 2.3. Randomization and Concealment

A simple randomization method with a 1:1 allocation ratio was used. The randomization list was generated using GraphPad QuickCalcs (online version; GraphPad Software, San Diego, CA, USA) by an independent individual who was not involved in any study procedures. The list was printed and used to prepare sealed, opaque envelopes to ensure allocation concealment. All envelopes were placed in a box, and after completion of the baseline assessment, each participant was asked to draw one envelope to determine assignment to either the MWM group or the sham MWM group. The outcome assessor remained blinded to group allocation throughout the study, and participants were unaware of whether they received the active or sham intervention.

### 2.4. Participants

Participants were adults aged 18 years or older with unilateral or bilateral knee OA diagnosed by orthopedic surgeons according to the American College of Rheumatology criteria and classified as Kellgren–Lawrence grade 2 or 3 and had a body mass index (BMI) between 18.5 and 30 kg/m^2^. Eligible participants reported knee pain greater than 3 on the Visual Analog Scale (VAS). Participants were excluded if they had undergone knee or lower-limb surgery within the previous six months; had significant joint diseases such as rheumatoid arthritis, gout, or other inflammatory arthropathies; had a recent acute infection; presented with contraindications to MWM techniques; or were unable to discontinue anti-inflammatory medications for the required period prior to baseline assessment.

### 2.5. Interventions

All interventions, including the MWM or sham MWM procedures and the strengthening exercise program, were delivered by licensed physical therapists with more than 10 years of clinical experience, twice per week for four weeks [24]. The MWM and sham MWM procedures were implemented according to the protocol described in a published study [25]. Adverse events were monitored throughout the intervention period and recorded at each treatment session by the treating therapist.

#### 2.5.1. MWM Group

In the supine position, the therapists applied passive tibial glides (medial or lateral glides in the frontal plane; anterior or posterior glides in the sagittal plane; and rotational glides in the transverse plane) while participants actively performed knee movements. Directional testing was first conducted in the frontal plane, followed by the sagittal plane and then the transverse plane. The glide that produced the greatest pain-free improvement in knee motion was selected. If more than one glide direction resulted in pain-free improvement, the effectiveness of each was further assessed under weight-bearing conditions (during a partial squat), and the glide that produced the greatest functional improvement was selected for treatment. The therapists applied the selected glide at mid-range while the participant performed active, pain-free knee flexion and extension, followed by end-range overpressure. Each session consisted of three sets of 10 repetitions.

#### 2.5.2. Sham MWM Group

The sham MWM group performed the same knee movements without the application of a therapeutic glide. Instead, the therapists’ hands were gently placed on the participants’ knees, with one hand on the tibia and the other on the femur, without applying pressure or joint glide. To simulate MWM, the therapists performed slight external rotation of the skin only, thereby mimicking the tactile sensation of true MWM without producing a mechanical effect. Sham movements were repeated for three sets of 10 repetitions.

#### 2.5.3. Both Groups

All participants received an education and strengthening exercise program consistent with current clinical guidelines [12]. Education was provided during the first session prior to the initiation of the exercise program and addressed OA, activity modification, weight management, and long-term self-management strategies. The strengthening program included knee flexion and extension exercises performed using a leg-curl machine (10 repetitions × 3 sets), with resistance progressively increased according to patient tolerance. Detailed intervention procedures, including initial load, progression criteria, and rest intervals, are provided in the Supplementary File S2.

### 2.6. Outcome Measures

All outcomes were assessed at baseline and after four weeks by two independent assessors who were blinded to group allocation. Both assessors had more than 5 years of physical therapy experience and underwent standardized training to ensure consistency and reliability of measurement procedures.

Primary Outcome Measures

Blood samples were collected by a certified laboratory technician to measure CRP and ESR. CRP was analyzed using the Roche Hitachi Cobas C system (Roche Diagnostics, Mannheim, Germany), and ESR was measured using the Alifax Roller 20-PN analyzer (Polverara, Italy). Both biomarkers are commonly used and reliable clinical indicators of systemic inflammation [26]. Higher CRP and ESR values indicate greater inflammatory activity. Results were interpreted according to the certified laboratory reference ranges (CRP: 0–5 mg/L; ESR: 0–15 mm/h).

Secondary Outcome Measures

Pain intensity was assessed using a 10-cm VAS. Participants marked their perceived pain on a horizontal line ranging from 0 (no pain) to 10 (worst pain), and the score was recorded in centimeters. The VAS is a valid and reliable tool for assessing pain intensity in individuals with musculoskeletal conditions. Higher scores indicate greater pain intensity [27].

Functional status was measured using the Western Ontario and McMaster Universities Osteoarthritis Index (WOMAC), a valid and reliable 24-item questionnaire assessing pain (5 items; score range 0–20), stiffness (2 items; 0–8), and physical function (17 items; 0–68). Subscale scores were summed to obtain a total score [28], with higher WOMAC scores indicating greater pain, stiffness, and functional limitation. The validated Arabic version, which has shown acceptable reliability and validity in individuals with knee osteoarthritis, was used in this study [29].

Knee flexion range of motion (ROM) was measured in degrees using a universal manual goniometer following standardized positioning procedures. Goniometric measurement of knee ROM has demonstrated good validity and high intra- and inter-rater reliability in clinical settings [30]. Higher ROM values indicate greater knee joint mobility.

Strength of the knee flexor and extensor muscles was assessed using manual muscle testing (MMT) and graded according to the standard 0–5 ordinal scale [31]. A score of 0 indicates no visible muscle contraction, whereas a score of 5 indicates normal muscle strength against full resistance. MMT is a valid and reliable method for assessing muscle strength [32].

### 2.7. Statistical Analysis

Statistical analyses were performed using IBM SPSS Statistics version 25 (IBM Corp., Armonk, NY, USA). Continuous variables are presented as mean ± standard deviation (SD) or median (interquartile range) as appropriate, while categorical variables are expressed as frequencies and percentages.

Normality of continuous variables was assessed using the Shapiro–Wilk test. Baseline comparisons between groups were conducted using independent-samples *t*-tests for normally distributed variables, Mann–Whitney U tests for non-normally distributed variables, and chi-square tests for categorical variables.

Analysis of covariance (ANCOVA) was performed to compare post-intervention outcomes between groups, with baseline values entered as covariates and treatment group as the fixed factor. This approach was selected to adjust for potential baseline differences and improve statistical precision. Assumptions of ANCOVA were evaluated by examining the normality of residuals, homogeneity of variances (Levene’s test), and homogeneity of regression slopes. Outliers were assessed using boxplots, standardized residuals, and Cook’s distance. In cases where influential observations were identified, sensitivity analyses were conducted to assess the robustness of the results.

Within-group (pre–post) changes were evaluated as secondary analyses. For variables demonstrating non-normal distributions, logarithmic transformation was applied prior to analysis to improve distributional symmetry. Normality of difference scores (post–pre) was then assessed using the Shapiro–Wilk test and visual inspection of Q–Q plots. For outcomes with normally distributed difference scores, a one-way repeated measures analysis of variance (ANOVA) with two time points (pre and post) was used to assess changes over time within each group. Mean differences were reported with 95% confidence intervals (CIs). For outcomes violating the normality assumption despite transformation, the Wilcoxon signed-rank test was applied. Median differences were reported with corresponding 95% CIs. All analyses were conducted according to the intention-to-treat (ITT) principle. Missing post-intervention data were handled using the last observation carried forward (LOCF) method [33]. Additionally, sensitivity analyses using complete-case data were performed to evaluate the stability of the findings. No interim analyses or stopping guidelines were planned because of the short duration of the trial. Statistical significance was set at *p* < 0.05.

## 3. Results

### 3.1. Participant Flow and Baseline Characteristics

**Figure 1:**
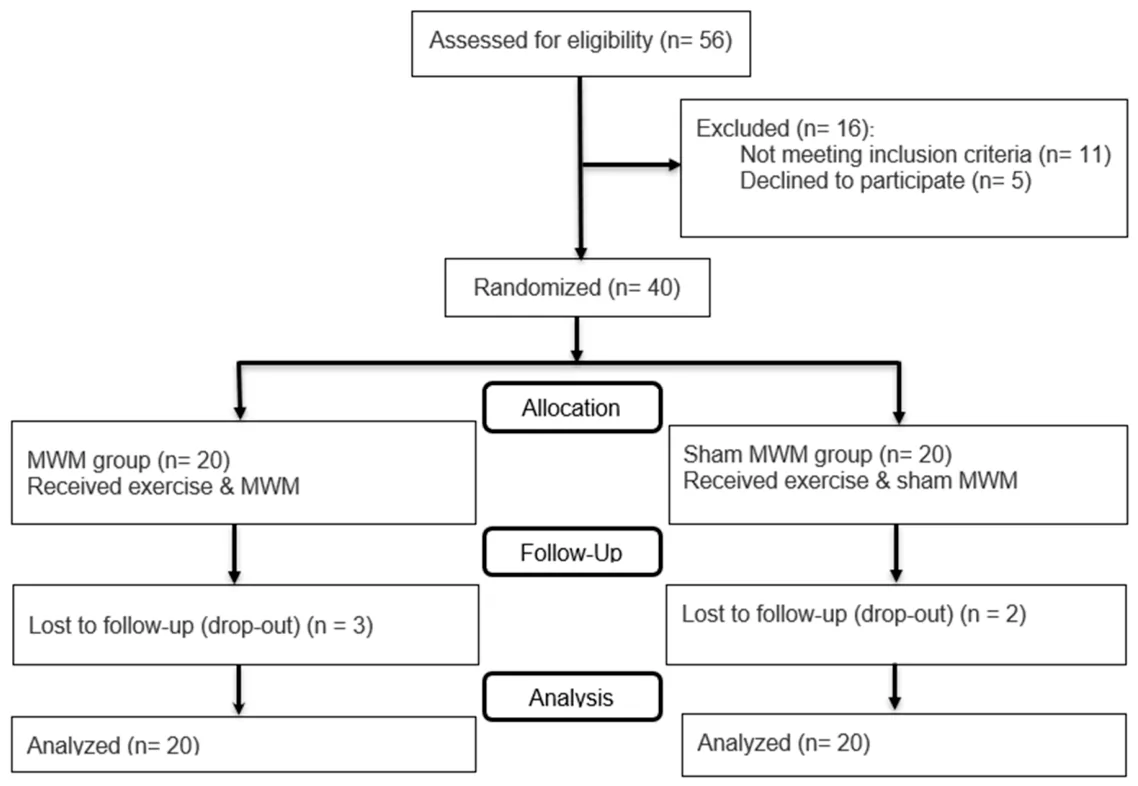
CONSORT flow diagram of participant enrollment and randomization. MWM, mobilization with movement.

Fifty-six participants were assessed for eligibility. Of these, 40 met the inclusion criteria and were randomly allocated to either the MWM group or the sham MWM group (Figure 1). Participants were recruited and followed for outcome assessment between May 2025 and November 2025. Participants received the allocated interventions as planned: the MWM group received mobilization with movement in addition to the standardized exercise and education program, whereas the sham MWM group received sham mobilization with movement in addition to the same standardized exercise and education program. Participants who completed the trial attended a mean of 6.7 of the 8 scheduled sessions (84%).

Both groups were homogeneous in demographic characteristics at baseline (Table 1). The mean age of participants was approximately 51 years (range: 31–73 years), and they were classified as overweight (median BMI: 28.0 kg/m^2^). The duration of their pain was chronic, with median durations of 30 months in the MWM group and 36 months in the sham MWM group. The left knee was more frequently affected than the right, with most participants having grade II knee OA. No adverse events or unintended effects were reported in either group during the intervention period.

Table 2 demonstrates baseline values and within-group changes of the primary and secondary outcomes for both groups. Most baseline variables were comparable between groups (*p* > 0.05). However, higher VAS and WOMAC scores and lower ROM were observed in the MWM group. Baseline values of outcome variables were therefore entered as covariates in the ANCOVA models.

### 3.2. Effects of the Interventions on Primary Outcomes

For biomarkers, both MWM and Sham MWM groups demonstrated significant reductions after treatment compared to baseline in CRP (MWM: mean difference [MD] = −0.3 ln mg/L, 95% CI = −0.586, −0.046, *p* = 0.024; Sham MWM: MD = −0.5 ln mg/L, 95% CI = −0.775, −0.141, *p* = 0.007). However, no significant changes were observed in ESR (MWM: MD = 0.0 mm/h, 95% CI = −3.000, 2.000, *p* = 0.756; Sham MWM: MD = −2.0 mm/h, 95% CI = −7.000, 0.000, *p* = 0.148) (Table 2). No significant between-group differences were observed for CRP (MD = −0.13 mg/L, 95% CI = −2.873, 2.620, *p* = 0.926) or ESR (MD = 3.45 mm/h, 95% CI = −2.600, 9.492, *p* = 0.256) (Table 3).

### 3.3. Effects of the Interventions on Secondary Outcomes

Compared to baseline, significant within-group improvements were observed in both groups across all secondary outcomes, namely VAS (MWM: MD = −3.6 cm, 95% CI = −4.450, −1.600, *p* < 0.001; Sham MWM: MD = −3.0 cm, 95% CI = −4.500, −1.650, *p* < 0.001), WOMAC (MWM: MD = −19.9 points, 95% CI = −29.133, −10.567, *p* < 0.001; Sham MWM: MD = −13.3 points, 95% CI = −19.516, −6.984, *p* < 0.001), knee flexion ROM (MWM: MD = 17.5°, 95% CI = 0.000, 23.500, *p* = 0.001; Sham MWM: MD = 5.0°, 95% CI = 0.000, 5.000, *p* = 0.026), knee flexor strength (MWM: MD = 1.0 grade, 95% CI = 0.000, 1.000, *p* = 0.001; Sham MWM: MD = 0.5 grade, 95% CI = 0.000, 1.000, *p* = 0.002), and knee extensor strength (MWM: MD = 1.0 grade, 95% CI = 0.000, 1.000, *p* = 0.005; Sham MWM: MD = 0.5 grade, 95% CI = 0.000, 1.000, *p* = 0.002) (Table 2).

On the other hand, ANCOVA adjusting for baseline values revealed no significant between-group differences for VAS (MD = 0.620 cm, 95% CI = −1.018, 2.256, *p* = 0.449), WOMAC (MD = −1.370 points, 95% CI = −12.035, 9.292, *p* = 0.796), knee flexor strength (MD = 0.130 grade, 95% CI = −0.233, 0.479, *p* = 0.463), or knee extensor strength (MD = 0.010 grade, 95% CI = −0.340, 0.371, *p* = 0.935). However, a significant between-group difference was observed for knee flexion ROM in favor of the MWM group (MD = 6.570°, 95% CI = 0.138, 12.998, *p* = 0.046) (Table 3).

### 3.4. Sensitivity Analyses

Complete-case sensitivity analyses produced findings consistent with the intention-to-treat analyses. No subgroup or other ancillary analyses were performed.

## 4. Discussion

This RCT investigated the effects of MWM combined with standard care versus sham MWM plus standard care on inflammatory biomarkers (CRP and ESR), pain (VAS), functional status (WOMAC), knee flexion ROM measured with a universal goniometer, and strength of the knee flexor and extensor muscles assessed using MMT in individuals with knee OA. Within-group analysis showed significant reductions in CRP in both groups, whereas ESR did not change significantly. In addition, both groups demonstrated significant improvements in pain, functional status, knee flexion ROM, and knee flexor and extensor muscle strength. However, adding MWM to standard care did not result in superior improvements compared with Sham MWM in inflammatory biomarkers, pain, functional status, or knee muscle strength. The only between-group difference observed was in knee flexion ROM, which favored the MWM group.

The reductions in CRP observed in both groups may reflect the anti-inflammatory effects of the standardized exercise program rather than MWM specifically, consistent with evidence that exercise reduces circulating CRP in patients with knee OA [34,35]. These findings partially contrast with the comparable manual therapy RCT that examined Maitland mobilization compared with structured exercise in 70 patients with knee OA. At 6 weeks, a significant between-group difference in CRP favoring the Maitland mobilization group was reported, alongside significant within-group improvements in both groups [16]. However, several methodological differences limit direct comparison. Most notably, the intervention in the study by Shabbir et al. was delivered in 18 treatment sessions over six weeks, whereas the present study delivered only 8 sessions over four weeks. This higher number of treatment sessions may explain the detection of between-group differences in CRP. This interpretation is supported by a recent systematic review and meta-analysis demonstrating that greater treatment intensity was associated with larger reductions in CRP levels in individuals with knee OA [36].

The absence of ESR change in either group requires careful interpretation. ESR is a non-specific inflammatory marker influenced by multiple confounding variables, including age, sex, anemia, and plasma protein composition, which may reduce its sensitivity to detect short-term intervention-induced changes [37]. This lack of ESR responsiveness is not unique to the present RCT. Harpham et al., in a systematic review and meta-analysis of aerobic exercise interventions in individuals with ankylosing spondylitis, reported significant reductions in CRP without corresponding significant changes in ESR [38]. However, reductions in both CRP and ESR have been reported in some exercise-based studies involving patients with rheumatoid arthritis [39,40]. These mixed findings of the effectiveness of non-pharmacological interventions on ESR may depend on factors such as disease characteristics and intervention duration.

All secondary outcome measures (VAS, WOMAC, knee ROM, and knee muscle strength) improved meaningfully in both MWM and Sham MWM groups. However, no significant between-group differences were observed after ANCOVA adjustment. These findings might suggest that the shared supervised exercise program likely accounted for most of the symptomatic improvement, consistent with current clinical practice guidelines that consider exercise as the core component of knee OA management [11,12]. In contrast, Alkhawajah and Alshami reported superior short-term improvements in pain and related clinical outcomes following MWM compared with sham MWM. However, their study assessed only the immediate and short-term effects of MWM without a concurrent structured exercise program in both groups [25]. Differences in treatment context, comparator design, and follow-up duration across studies may explain the variability in reported between-group effects. Similarly, Shabbir et al. compared Maitland mobilization delivered three times weekly with a conventional physical therapy program [16], resulting in a different treatment contrast than the present study, which evaluated the additive effect of MWM alongside exercise in both groups.

The between-group advantage in knee flexion ROM was the only significant between-group finding and may be of potential clinical relevance. After adjusting for a significant baseline difference of approximately 12.5° in favor of the sham MWM group, MWM still produced a significantly greater improvement in flexion. The magnitude of change exceeded recently established minimal clinically important change (MCIC) thresholds for knee flexion (3.8–6.4°) [41]. However, as these thresholds were derived from within-group change, they provide only an approximate benchmark for a between-group difference, and the clinical relevance of this effect should be interpreted with caution. Mechanistically, this finding is coherent: MWM applies a sustained accessory tibial glide during active movement, which may address arthrokinematic restrictions that limit flexion range [42]. The persistence of statistical significance despite conservative ANCOVA adjustment further strengthens confidence in this result and aligns with prior evidence supporting MWM for ROM restoration in knee OA [18,25].

No significant between-group differences were observed in knee flexor or extensor muscle strength, which is unsurprising given that both groups performed an identical resistance training program. The absence of differential effects may also reflect limitations of the MMT grading scale, which has been shown to have limited sensitivity compared with quantitative dynamometry, particularly for detecting subtle strength changes at higher force levels [43]. Future trials should incorporate objective dynamometric assessments to better evaluate potential neuromuscular adaptations associated with MWM.

This study has several strengths. It employed a randomized controlled design with concealed allocation and statistical adjustment for baseline imbalance using ANCOVA, enhancing internal validity. The inclusion of a sham manual therapy condition allowed partial control of contextual and therapist-related effects, thereby strengthening the interpretability of between-group comparisons. Additionally, outcomes were assessed using validated clinical measures with established minimal clinically important improvement thresholds, facilitating interpretation of clinical relevance.

Several limitations should be acknowledged. The modest sample size and relatively short intervention duration may have limited the ability to detect longer-term additive effects of MWM beyond exercise. A significant baseline imbalance in pain and function scores, although statistically adjusted, may have influenced effect estimates. Muscle strength was assessed using manual muscle testing, which has limited sensitivity compared with quantitative dynamometry and may not detect subtle neuromuscular adaptations. Future studies assessing inflammatory mediators such as IL-1β, IL-6, and TNF-α locally in synovial fluid may complement systemic markers such as CRP and ESR by providing a more precise characterization of joint-level inflammation. Finally, the absence of long-term follow-up precludes conclusions regarding durability of treatment effects.

## 5. Conclusions

In individuals with knee OA, the addition of MWM to a structured exercise program did not result in superior improvements in the primary inflammatory markers (CRP and ESR) compared with sham MWM plus exercise over the short term. Similarly, no between-group differences were observed in pain, function, or muscle strength. However, MWM demonstrated a statistically significant advantage in knee flexion ROM, which may be of potential clinical relevance. These findings reinforce exercise as the cornerstone of knee OA management while suggesting that MWM may provide targeted benefits for restoring joint mobility. Further research with longer follow-up and more sensitive outcome measures is warranted to clarify the potential mechanistic and long-term effects of this intervention.

## Figures and Tables

**Table 1:** Characteristics of participants in both groups at baseline.

Variable	MWM (*n* = 20)	Sham MWM (*n* = 20)	*p*-Value
Age (years) *	51.4 ± 10.3	51.4 ± 13.60	0.990
BMI (kg/m^2^) #	27.9 (3.0)	27.5 (5.2)	0.465
Symptom Duration (months) #	30.0 (40.3)	36.0 (55.3)	0.643
Gender (male/female) $	9 (45%)/11 (55%)	8 (40%)/12 (60%)	1.000
Affected knee (right/left) $	9 (45%)/11 (55%)	6 (30%)/14 (70%)	0.514
K&L knee OA grade (2/3) $	15 (75%)/5 (25%)	15 (75%)/5 (25%)	1.000

Data are expressed as mean ± standard deviation (*), median and interquartile range (#), and frequency and percentage ($). BMI, body mass index; K&L, Kellgren–Lawrence grading system; MWM, mobilization with movement; OA, osteoarthritis.

**Table 2:** Baseline and within-group differences in the primary and secondary outcome measures for both groups.

Variable	Group	Baseline Mean ± SD/Median (IQR)	Post-Intervention Mean ± SD/Median (IQR)	Within-Group Differences Mean/Median Difference (95% CI)	Within-Group *p*-Value	Baseline *p*-Value
CRP (mg/L) *	MWM	1.2 ± 1.1	0.9 ± 1.2	−0.3 (−0.586, −0.046)	0.024	
	Sham MWM	1.6 ± 0.9	1.1 ± 1.0	−0.5 (−0.775, −0.141)	0.007	0.240
ESR (mm/h)	MWM	13.0 (17.0)	17.50 (16.5)	0.0 (−3.000, 2.000)	0.756	
	Sham MWM	18.0 (21.4)	17.50 (17.5)	−2.0 (−7.000, 0.000)	0.148	0.304
VAS (10 cm)	MWM	7.9 (1.6)	4.9 (2.4)	−3.6 (−4.450, −1.600)	<0.001	
	Sham MWM	5.4 (2.3)	1.9 (3. 2)	−3.0 (−4.500, −1.650)	<0.001	0.002
WOMAC (/96)	MWM	50.9 ± 22.0	30.6 ± 19.0	−19.9 (−29.133, −10.567)	<0.001	
	Sham MWM	36.6 ± 15.1	23.3 ± 19.7	−13.3 (−19.516, −6.984)	<0.001	0.025
Knee Flexion ROM (°)	MWM	117.5 (24.0)	135.0 (11.3)	17.5 (0.000, 23.500)	0.001	
	Sham MWM	130.0 (10.0)	132.5 (5.0)	5.0 (0.000, 5.000)	0.026	0.002
Knee Flexor strength (/5)	MWM	4.0 (1.0)	5.0 (1.0)	1.0 (0.000, 1.000)	0.001	
	Sham MWM	4.0 (0.0)	5.0 (1.0)	0.5 (0.000, 1.000)	0.002	0.168
Knee Extensor strength (/5)	MWM	4.0 (1.0)	5.0 (1.0)	1.0 (0.000, 1.000)	0.005	
	Sham MWM	4.0 (0.3)	5.0 (1.0)	0.5 (0.000, 1.000)	0.002	0.060

CI, confidence interval; CRP, C-reactive protein; ESR, erythrocyte sedimentation rate; MWM, mobilization with movement; ROM, range of motion; SD, standard deviation; VAS, Visual Analog Scale; WOMAC, Western Ontario and McMaster Universities Osteoarthritis Index; *, values are presented after natural logarithmic transformation; °, degree.

**Table 3:** Between-group differences in the primary and secondary outcome measures.

Variable	Group	Adjusted Mean (95% CI)	Adjusted Mean Difference (95% CI)	*p*-Value	Partial η^2^	F
CRP (mg/L)	MWM	4.47 (2.530, 6.405)	−0.13 (−2.873, 2.620)	0.926	<0.001	0.009
	Sham MWM	4.59 (2.657, 6.531)				
ESR (mm/h)	MWM	20.92 (16.663, 25.183)	3.45 (−2.600, 9.492)	0.256	0.035	0.251
	Sham MWM	17.48 (13.217, 21.737)				
VAS (10 cm)	MWM	4.06 (2.977, 5.147)	0.62 (−1.018, 2.256)	0.449	0.016	0.149
	Sham MWM	3.44 (2.358, 4.528)				
WOMAC (/96)	MWM	26.26 (18.964, 33.565)	−1.37 (−12.035, 9.292)	0.796	0.002	0.068
	Sham MWM	27.64 (20.335, 34.936)				
Knee Flexion ROM (°)	MWM	134.33 (130.088, 138.580)	6.57 (0.138, 12.998)	0.046	0.104	4.284
	Sham MWM	127.77 (123.520, 132.012)				
Knee Flexor Strength (/5)	MWM	4.59 (4.338, 4.840)	0.13 (−0.233, 0.479)	0.463	0.015	0.550
	Sham MWM	4.46 (4.206, 4.714)				
Knee Extensor Strength (/5)	MWM	4.66 (4.423, 4.912)	0.01 (−0.340, 0.371)	0.935	0.001	0.456
	Sham MWM	4.65 (4.408, 4.886)				

CI, confidence interval; CRP, C-reactive protein; ESR, erythrocyte sedimentation rate; MWM, mobilization with movement; ROM, range of motion; VAS, Visual Analog Scale; WOMAC, Western Ontario and McMaster Universities Osteoarthritis Index; °, degree.

## Data Availability

The data supporting the findings of this study are available from the corresponding author upon reasonable request, subject to the ethical and privacy restrictions of Jazan University Hospital.

## Supplementary Materials

The following supporting information can be downloaded at https://www.mdpi.com/article/10.3390/medicina62102016/s1, File S1: CONSORT checklist [44]; File S2: Intervention protocol.

## Abbreviations

The following abbreviations are used in this manuscript:

CONSORT	Consolidated Standards of Reporting Trials
CRP	C-reactive protein
ESR	Erythrocyte sedimentation rate
IRB	Institutional Review Board
MWM	Mobilization with movement
OA	Osteoarthritis
RCT	Randomized controlled trial
VAS	Visual Analog Scale
WOMAC	Western Ontario and McMaster Universities Osteoarthritis Index
