# Supplementary material for: Effects of Mobilization with Movement Versus Sham Mobilization on Inflammatory Biomarkers and Clinical Outcomes in Patients with Knee Osteoarthritis: A Randomized Double-Blind Clinical Trial
Source: Medicina (Kaunas). 2026 Oct 18;62(10):2016. doi: 10.3390/medicina62102016 (PMC13706732; doi:10.3390/medicina62102016)
Supplement: Supplementary file 1 [file medicina-62-02016-s001.zip › File_S2_Intervention_protocol.pdf]

Intervention protocols

1 .Patient Education

All participants received standardized education during the first treatment session before initiating the exercise program. The education component included the following:

a. A brief explanation of knee osteoarthritis, including the nature of the condition and common symptoms.

b. An explanation of the role of physical therapy in reducing pain and improving functional mobility and quality of life.

c. Emphasis on the importance of maintaining physical activity and avoiding a sedentary lifestyle.

d. Instructions to perform exercises within a tolerable pain range and to stop exercising and inform the therapist in the event of severe pain or increased swelling.

e. Explanation of the importance of strengthening the muscles surrounding the knee joint to support the joint, reduce pain, and improve function.

2 .Manual Therapy

2.1 .Mobilization-with-Movement Group

A sustained accessory glide was applied to the tibia during active knee flexion and extension.

Glide direction testing:

The optimal glide direction was determined individually for each participant. Glides were tested in the supine position in the following order: frontal plane glides (medial and lateral), sagittal plane glides (anterior and posterior), and rotational glides. The direction that most effectively reduced pain and improved range of motion was selected.

If the movement remained pain-free in the supine position, or if multiple glide directions were equally effective, testing was repeated in a weight-bearing position to confirm the optimal direction.

MWM application:

The therapist applied the selected glide force to the tibia while the knee was positioned at mid-range. The participant then actively flexed and extended the knee through the pain-free range of motion while the glide force was maintained. Overpressure was applied at the end range if the movement was pain-free.

Dosage:

The intervention was performed for 10 repetitions for 3 sets.

2.2 .Sham Mobilization-with-Movement Group

The therapist's hands were gently placed on the participant's knee, with one hand on the tibia and the other on the femur, without applying a mobilizing force or joint pressure.

The participant performed active knee flexion and extension movements. To simulate MWM, the therapist performed slight external rotation of the skin only, mimicking the tactile sensation of true MWM without influencing the underlying joint mechanics.

Dosage:

The sham intervention was performed for 10 repetitions for 3 sets.

3 .Standard Care: Knee Strengthening Exercises

All participants performed supervised resisted knee flexion and extension exercises in a seated position using a leg-curl device.

The resistance was determined according to participant tolerance. Exercise parameters were progressed as tolerated while maintaining the same exercise type.

Dosage and progression:

The initial load was set individually as the highest weight with which the participant could complete three sets of 10 repetitions with correct technique and without excessive fatigue or pain. If the participant could not complete the prescribed repetitions or showed signs of excessive fatigue, the load was reduced to the previous weight-stack level. In subsequent sessions, the load was progressively increased by one weight-stack increment when the participant could complete all three sets of 10 repetitions with correct technique and without excessive fatigue. Rest intervals of 60–120 seconds were provided between sets according to participant tolerance.

Supervision:

All exercises were supervised by the therapist to ensure correct technique. Exercise resistance was adjusted as needed according to participant tolerance, as described above.
